# Modeling and Assessment of GPS/BDS Combined Precise Point Positioning

**DOI:** 10.3390/s16071151

**Published:** 2016-07-22

**Authors:** Junping Chen, Jungang Wang, Yize Zhang, Sainan Yang, Qian Chen, Xiuqiang Gong

**Affiliations:** 1 Shanghai Astronomical Observatory, Chinese Academy of Sciences, Shanghai 200030, China; 1335465@tongji.edu.cn (J.W.); 1130797@tongji.edu.cn (Y.Z.); snyang@shao.ac.cn (S.Y.); qianchen@shao.ac.cn (Q.C.); xqgong@shao.ac.cn (X.G.); 2 Shanghai Key Laboratory of Space Navigation and Positioning Techniques, Shanghai Astronomical Observatory, Chinese Academy of Sciences, Shanghai 200030, China; 3 College of Surveying and Geo-informatics Engineering, Tongji University, Shanghai 200092, China

**Keywords:** multi-GNSS, BDS, precise point positioning, MGEX, ISB

## Abstract

Precise Point Positioning (PPP) technique enables stand-alone receivers to obtain cm-level positioning accuracy. Observations from multi-GNSS systems can augment users with improved positioning accuracy, reliability and availability. In this paper, we present and evaluate the GPS/BDS combined PPP models, including the traditional model and a simplified model, where the inter-system bias (ISB) is treated in different way. To evaluate the performance of combined GPS/BDS PPP, kinematic and static PPP positions are compared to the IGS daily estimates, where 1 month GPS/BDS data of 11 IGS Multi-GNSS Experiment (MGEX) stations are used. The results indicate apparent improvement of GPS/BDS combined PPP solutions in both static and kinematic cases, where much smaller standard deviations are presented in the magnitude distribution of coordinates RMS statistics. Comparisons between the traditional and simplified combined PPP models show no difference in coordinate estimations, and the inter system biases between the GPS/BDS system are assimilated into receiver clock, ambiguities and pseudo-range residuals accordingly.

## 1. Introduction

To prepare for the next phase of generating products for all GNSS available, the IGS [1] initiated the IGS Multi-GNSS Experiment (MGEX) campaign. The MGEX campaign focuses on tracking the newly available GNSS signals including the Chinese BeiDou Navigation Satellite System (BDS). BDS had been providing regional service since December 2012 with five Geostationary Earth Orbit (GEO) satellites, five Inclined Geosynchronous Satellite Orbit (IGSO) satellites and four Medium Earth Orbit (MEO) satellites. Many studies are being carried out and much progress has been achieved in BDS-only and GPS/BDS-combined precise data analysis. Precise BDS orbits/clocks are currently available at many institutes and several IGS analysis centers [2,3,4,5].

Many publications demonstrate the daily static Precise Point Positioning (PPP, [6] using BDS observations, and precise orbits/clocks could reach a precision of a few cm [5,7,8]. With the additional tracking of BDS constellations and therefore, a significant increase of observed satellites, GPS/BDS combined PPP could improve solution availability and accuracy by improving tracking geometry with a reduction of the position dilution of precision (PDOP) [9], especially in environments like urban canyons and ravines where there are limits in sky view. Chen et al. [10] demonstrated that near real time GPS/BDS combined PPP solution show a higher degree of precision and better robustness, which is very important for tsunami early warning. Also, for GNSS-based precipitable water vapor retrieval, GPS/BDS combined PPP could obtain more accurate and reliable water vapor estimates than GPS-only or BDS-only PPP [11]. Guo et al. [12] evaluated the orbit and clock of different GNSS systems from different analysis centers comprehensively and has shown the accuracy improvement of Mulit-GNSS PPP rather than single-system PPP.

Compared to the GPS-only and BDS-only PPP, the key issue in GPS/BDS PPP is the handling of inter system bias (ISB). Station-wise GPS/BDS inter-system bias includes two parts, one is the difference between system time GPST and BDT, and the other comes from the station-wise instrument hardware delay difference when tracking satellites of different systems. For most geodetic receivers and antennas, the instrument hardware delay is not calibrated and it will be assimilated into the receiver clock. Thus, the GPS/BDS inter-system bias shows up as the receiver clock differences between GPS-only and BDS-only PPP. For GPS/BDS combined PPP, two kinds of parameterization strategies are widely implement: (1) two receiver clock parameters at each epoch; (2) epoch-wise receiver clock and ISB parameter. Since for one station, the difference of hardware delay between different satellite systems is over one day interval [13,14,15], ISB is usually treated as a daily constant [9,16,17,18]. Chen et al. [19] developed a new simplified multi-GNSS PPP model, which does not include ISB parameter and observations of different GNSS systems could be treated in a unique way. Jiang et al. [20] demonstrated that a priori constraint of predicted ISB in GPS/BDS combined PPP could shorten convergence time and also, improve positioning accuracy slightly. Furthermore, many studies have been carried out over the handling of ISB in multi-GNSS data processing, including GPS/BDS inter-ambiguity resolution [21], GPS/BDS single-frequency short baseline RTK [22,23] and long baseline relative positioning [24], GPS/BDS/GLONASS/Galieo/QZSS ISBs analysis with different types of receivers [25], BDS/Galileo/QZSS and GPS single-frequency RTK [26].

In this paper, we present and evaluate GPS/BDS PPP models, where single system and combined PPP models are discussed. In the following, Section 2 presents the traditional and simplified GPS/BDS PPP model; Section 3 presents data analysis and compares daily positioning qualities; Section 4 compares the solved parameters between the traditional and simplified combined PPP models; finally, Section 5 summarizes the main points of this paper.

## 2. GPS/BDS Combined PPP Models

### 2.1. PPP Model for Single System

Taking a GPS system as an example, pseudo-range and phase observation functions of the ionosphere-free combination between a receiver and a satellite G can be written as:

(1) $$\begin{array}{l} P^{G}=\rho^{G}+c\cdot dt_{G}+b_{G}+ZPD^{G}+\varsigma^{G}\\ L^{G}=\rho^{G}+c\cdot dt_{G}+B_{G}+N^{G}+ZPD^{G}+\varepsilon^{G} \end{array}$$

where $P^{G},L^{G}$ are the pseudo-range and carrier phase observation; $\rho^{G}$ is geometrical distance; c is light speed and $c\cdot dt_{G}$ is receiver clock correction; $b_{G}$ and $B_{G}$ are receivers pseudo-range and carrier phase hardware delay bias; $N^{G}$ is ambiguity, and $ZPD^{G}$ is the slant tropospheric delay, $\varsigma^{G}$ and $\varepsilon^{G}$ are noise terms.

The pseudo-range hardware delay biases $b_{G}$ is assimilated into clock correction $c\cdot dt_{G}$. The carrier phase hardware delay biases $B_{G}$ is satellite dependent and stable over time, and it will be grouped into ambiguity [27,28]. Thus, the ionosphere-free combination PPP observation can be rewritten as:

(2) $$\begin{array}{l} P^{G}=\rho^{G}+c\cdot d{\bar{t}}_{G}+ZPD^{G}+\varsigma^{G}\\ L^{G}=\rho^{G}+c\cdot d{\bar{t}}_{G}+{\bar{N}}^{G}+ZPD^{G}+\varepsilon^{G} \end{array}$$

where $d{\bar{t}}_{G}$ and ${\bar{N}}^{G}$ are reformed station clock and ambiguity with:

(3) $$\begin{array}{l} c\cdot d{\bar{t}}_{G}=c\cdot dt_{G}+b_{G}\\ {\bar{N}}^{G}=N^{G}+B_{G}-b_{G} \end{array}$$

For BDS phase and pseudo-range observations the same Equations (2) and (3) also apply.

### 2.2. Traditional GPS/BDS Combined PPP Model

The ionosphere-free pseudo-range and phase observations for the GPS/BDS combined PPP can be written as:

(4) $$\begin{array}{l} P^{G}=\rho^{G}+c\cdot d{\bar{t}}_{G}+ZPD^{G}+\varsigma^{G}\\ L^{G}=\rho^{G}+c\cdot d{\bar{t}}_{G}+{\bar{N}}^{G}+ZPD^{G}+\varepsilon^{G}\\ P^{C}=\rho^{C}+c\cdot d{\bar{t}}_{C}+ZPD^{C}+\varsigma^{C}\\ L^{C}=\rho^{C}+c\cdot d{\bar{t}}_{C}+{\bar{N}}^{C}+ZPD^{C}+\varepsilon^{C} \end{array}$$

In Equation (4), the superscript and subscript G,C refers to GPS and BDS satellites. The slant tropospheric delay term ZPD could be modelled as the hydrostatic and wet parts with following:

(5) $$ZPD=mf_{h}\cdot ZHD+mf_{w}\cdot ZWD$$

In Equation (5), ZHD,ZWD are the hydrostatic and wet zenith path delays, $mf_{h},mf_{w}$ are the mapping functions. The hydrostatic part of ZPD is normally corrected using empirical models and the term $ZTD_{w}$ is estimated.

The traditional GPS/BDS combined PPP model requires the estimation of an additional inter-system bias parameter. Defining GPS as the reference system, the inter-system bias could be written as,

(6) $$\begin{array}{l} ISB=c\cdot d{\bar{t}}_{C}-c\cdot d{\bar{t}}_{G}\\ =c\cdot dt_{C}-c\cdot dt_{G}+b_{C}-b_{G}\\ =c\cdot \Delta dt+\Delta b \end{array}$$

Considering Equations (5) and (6), the traditional GPS/BDS combined PPP equations are written as,

(7) $$\begin{array}{l} P^{G}=\rho^{G}+c\cdot d{\bar{t}}_{G}+mf_{w}^{G}\cdot ZWD+\varsigma^{G}\\ L^{G}=\rho^{G}+c\cdot d{\bar{t}}_{G}+{\bar{N}}^{G}+mf_{w}^{G}\cdot ZWD+\varepsilon^{G}\\ P^{C}=\rho^{C}+c\cdot d{\bar{t}}_{G}+ISB+mf_{w}^{C}\cdot ZWD+\varsigma^{C}\\ L^{C}=\rho^{C}+c\cdot d{\bar{t}}_{G}+ISB+{\bar{N}}^{C}+mf_{w}^{C}\cdot ZWD+\varepsilon^{C} \end{array}$$

### 2.3. Simplified GPS/BDS Combined PPP Model

Based on the scaled sensitivity matrix (SSM, [19,29]) method, Chen et al. [19] proves that there is no correlation between ISB and coordinate parameters in multi-GNSS combined PPP, and ISB parameter can be removed in multi-GNSS PPP. Based on this conclusion, we remove the ISB term in Equation (7) and rewrite observation equations as:

(8) $$\begin{array}{l} P^{G}=\rho^{G}+c\cdot d{\bar{t}}^{\prime }+mf_{w}^{G}\cdot ZWD+\varsigma^{G^{\prime }}\\ L^{G}=\rho^{G}+c\cdot d{\bar{t}}^{\prime }+{\bar{N}}^{G^{\prime }}+mf_{w}^{G}\cdot ZWD+\varepsilon^{G}\\ P^{C}=\rho^{C}+c\cdot d{\bar{t}}^{\prime }+mf_{w}^{C}\cdot ZWD+\varsigma^{C^{\prime }}\\ L^{C}=\rho^{C}+c\cdot d{\bar{t}}^{\prime }+{\bar{N}}^{C^{\prime }}+mf_{w}^{C}\cdot ZWD+\varepsilon^{C} \end{array}$$

where $d{\bar{t}}^{\prime }$ is defined as new receiver clock, ${\bar{N}}^{G^{\prime }}$ and ${\bar{N}}^{C^{\prime }}$ are new ambiguity terms. Compared to Equation (7), the ISB term is absorbed in the new terms and pseudo-range residuals with:

(9) $$\begin{array}{l} d{\bar{t}}^{\prime }=d{\bar{t}}_{G}+\lambda \cdot ISB\\ {\bar{N}}^{G^{\prime }}={\bar{N}}^{G}-\lambda \cdot ISB\\ \varsigma^{G^{\prime }}=\varsigma^{G}-\lambda \cdot ISB\\ \varsigma^{C^{\prime }}=\varsigma^{C}+\left(1-\lambda \right)\cdot ISB\\ {\bar{N}}^{C^{\prime }}={\bar{N}}^{C}+\left(1-\lambda \right)\cdot ISB \end{array}$$

where λ is called the element of the SSM, which quantitatively defines the ratio of ISB parameter assimilated into the receiver clock.

As described in Chen et al. [19], the benefits of the new model are: (1) under some extreme circumstances, the traditional Multi-GNSS PPP model may final when there are only four satellites observed, while the new approach will still give coordinate solution; (2) the correlation between the clock and ambiguity parameters is reduced, thus making solution more stable using the new model; (3) Multi-GNSS PPP realization is simplified and unified. In this new model, observations of different GNSS systems are treated in a unified way as they were of the same satellite system. In this simplified model, the ISB is assimilated into a receiver clock and ambiguity parameter. Due to the much smaller weight assigned on pseudo-range observations, all pseudo-range observations will show big residuals. The GPS pseudo-range residuals are close to the amount of ISB value that assimilates into station clock parameter. The BDS pseudo-range residuals are close to the amount of ISB value that assimilates into BDS ambiguity.

## 3. Data Processing

**Figure 1:**
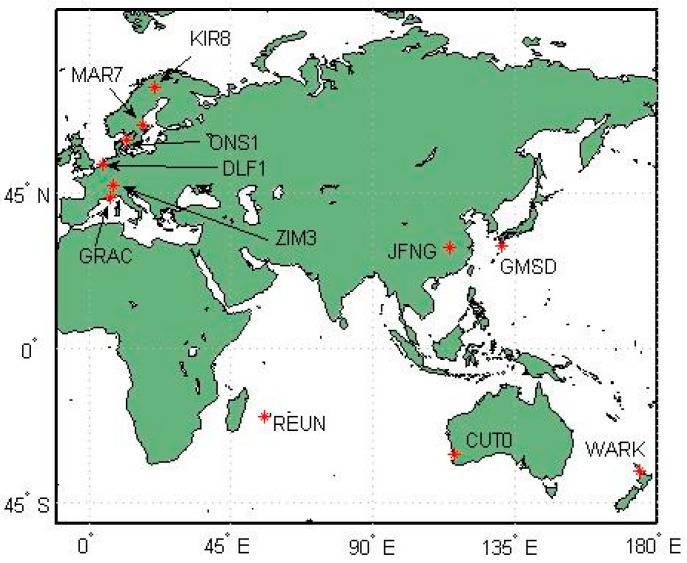
The geographic distribution of GPS/BDS stations used in the data analysis.

To assess GPS/BDS PPP performance under different processing models, and to validate the simplified GPS/BDS combined PPP model, daily static and kinematic PPP data analysis is performed. Data of 11 IGS MGEX stations in January 2014 is used. Figure 1 shows the geographic distribution of these tracking stations.

For results evaluation and comparison, data processing is performed in the following four scenarios: GPS-only PPP, BDS-only PPP, and GPS/BDS combined PPP with traditional and simplified models, where station-wise ISB is estimated as a daily constant in a traditional model.

Precise satellite orbits and satellite clocks from SHA [17,30] are used for data analysis. We apply the IGS absolute antenna phase center model for GPS observations [31], the phase-wind up modeling [32] and the station displacement are modeled according to the IERS conventions 2010 [33]. A cut-off angle of 7° is set for usable measurements and data sampling is set to 30 s. An elevation-dependent weighting strategy is applied to measurements at low elevations and the noise ratio between pseudo-range and phase observation is 500:1. Moreover, we estimate wet zenith path delay every hour by applying the most recently developed GPT2 empirical slant delay model [34]. An improved version of the LTW_BS software is used [35].

### 3.1. Position Differences between Static PPP and IGS Daily Solutions

**Figure 2:**
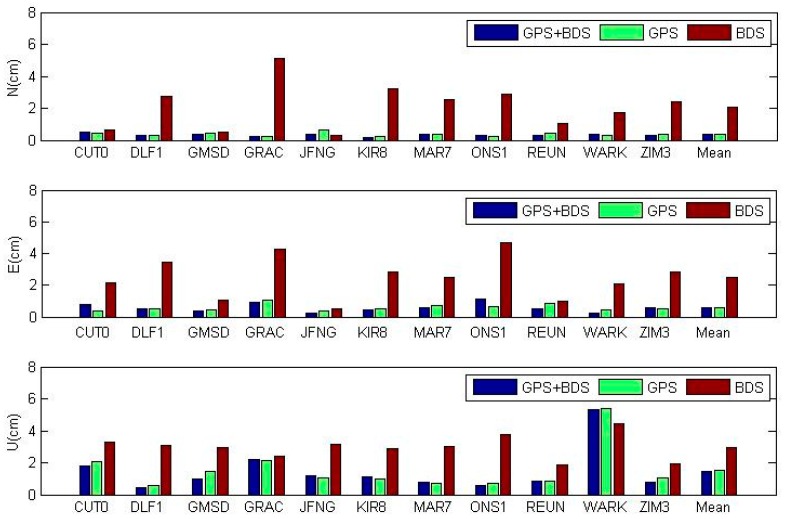
RMS of coordinate differences between daily static PPP and IGS daily solutions for each station in the North, East, and Up component, where PPP in different scenarios is shown in different color. The last column of each subplot is the mean value of the 11 stations.

We compared GPS/BDS daily position estimates with the IGS daily solutions covering the same period through a seven-parameter Helmert transformation [28,36]. The RMS of each station is showed in Figure 2 and the mean value is shown in Table 1. GPS/BDS combined PPP achieves the best performance compared with GPS-only PPP and BDS-only PPP. The improvement of combined PPP over GPS-only PPP is not very obvious. BDS-only PPP has the worst accuracy, which may be due to the less accurate BDS orbit and clock products and a regional satellite constellation with less satellites compared with GPS. We notice that stations JFNG (Wuhan, China), CUT0 (Perth, Australia), GMSD (Guts Masda, Japan), and REUN (Le Tampon, La Reunion, France) have the best accuracy in BDS PPP, which is due to the fact that more BDS satellites are tracked for these sites under the current BDS constellation. Also, the GPS/BDS combined PPP using a simplified and traditional model is nearly the same and their values differ at the level of µm, which will be discussed in detail in Section 3.3 and Section 4.

**Figure 3:**
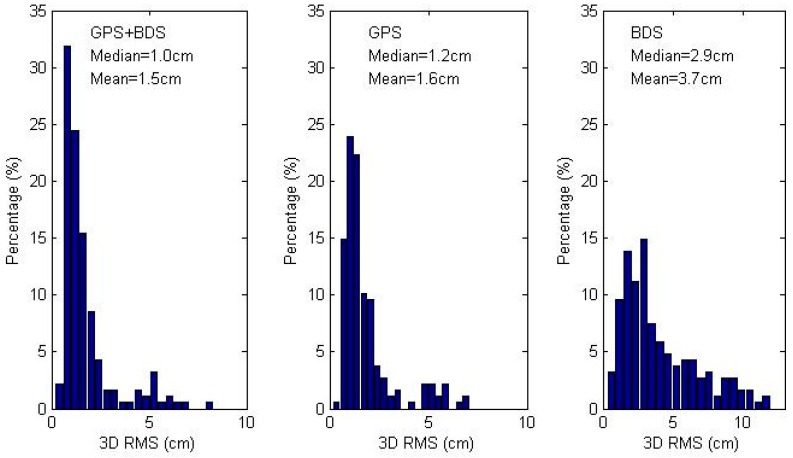
Magnitude distribution of 3D RMS of coordinate differences between daily static PPP and IGS daily solutions. Subplot presents the GPS/BDS combined, GPS-only and BDS-only solutions from left to right, respectively. The text of each subplot shows the median and mean value of 3D RMS.

We make 3D RMS statistics of the 11 stations over the whole month and plot the histogram of the three scenarios in Figure 3. The GPS/BDS combined PPP achieves the best accuracy, with a mean 3D RMS of 1.5 cm, while those of GPS-only and BDS-only is 1.6 cm and 3.7 cm, respectively. The percentage of 3D RMS within the range of [0, 1.5] cm is 26%, 40%, and 59% for BDS-only, GPS-only and combined solutions, which demonstrates a more accurate solution is obtained by adding observations from different systems.

### 3.2. Position Differences between Kinematic PPP and IGS Daily Solutions

**Figure 4:**
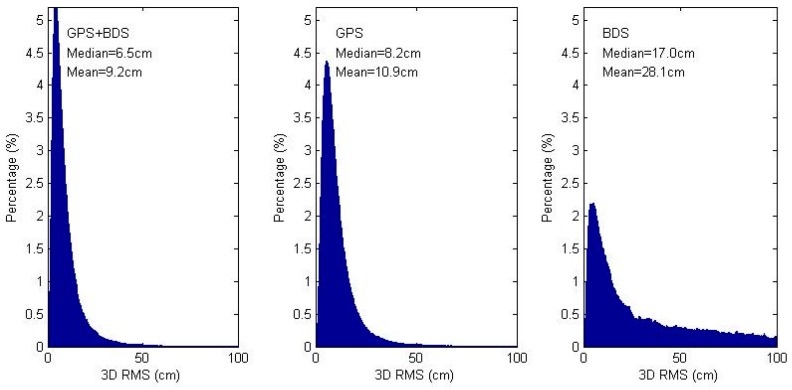
Magnitude distribution of 3D RMS of coordinate differences between epoch-wise kinematic PPP and IGS daily solutions. Subplot presents the GPS/BDS combined, GPS-only and BDS-only solutions from left to right, respectively. The texts of each subplot shows the median and mean 3D RMS.

To assess kinematic position estimates differences among PPP scenarios, epoch-wise kinematic coordinate estimates are compared with the IGS daily estimates. In each daily kinematic solution, the epoch-wise coordinates after the first 1 h (120 epochs) are used for statistics analysis. Figure 4 shows for all 11 stations the magnitude distribution of 3D RMS of kinematic coordinate differences. The mean 3D RMS are of 28.1, 10.9, 9.2 cm and median 3D RMS are of 17.0, 8.2, 6.5 cm for the BDS-only, GPS-only and GPS/BDS combined solutions. The magnitude distribution also shows a higher percentage of more accurate estimations in GPS/BDS combined solutions.

**Figure 5:**
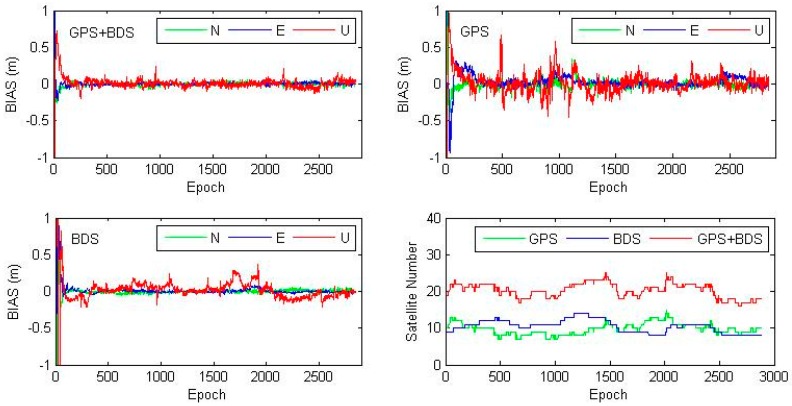
Epoch-wise kinematic PPP coordinate bias of GPS/BDS combined, GPS-only and BDS-only solutions in the North, East and Up component. Station: JFNG, DOY 028, 2014. Bottom-right subplot shows the number of satellites tracked at each epoch.

Kinematic PPP of station JFNG on DOY (Day of Year) 028, 2014 is shown in Figure 5. It shows that combined GPS/BDS kinematic PPP has much better stability and shorter convergence, while both GPS-only and BDS-only kinematic PPP show big scatters as large as 0.5 m. The number of tracked satellites is shown in the bottom-right subfigure of Figure 5, where number of available satellites of GPS/BDS combination almost doubles that of GPS-only and BDS-only cases. Statistics show that for JFNG, GPS-only kinematic PPP has a better accuracy than that of BDS, even if BDS-only kinematic PPP occasionally performs better that GPS when BDS has better satellite coverage with observations of good quality, such as JFNG at DOY 028, 2014.

### 3.3. Position Differences between Traditional and New Model

Assessing the differences between the position estimates can directly illustrate to what extent the traditional and simplified models agree in their positioning results. In the following we compare the GPS/BDS combined static PPP results between the traditional and simplified models.

**Figure 6:**
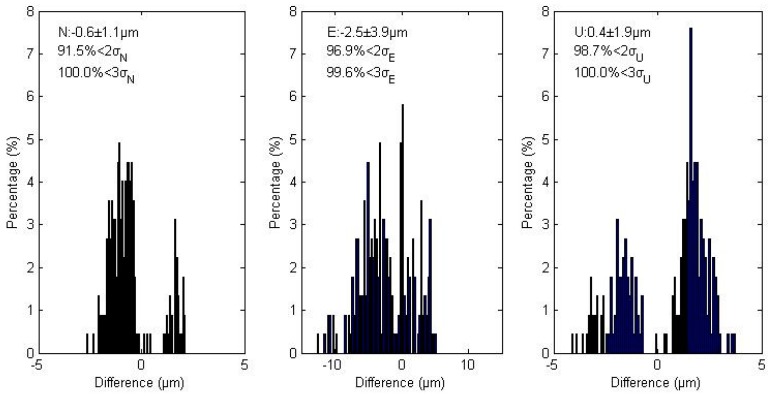
Magnitude distribution of all position differences of GPS/BDS combined static PPP between the traditional and simplified models in the North, East, and Up components. All subplots exhibit normal distributions. The top-left corner of each subplot shows the bias and the standard deviation (σ), as well as the percentages of deviations that are within 2σ and 3σ.

For each of the 11 IGS MGEX stations, we computed GPS/BDS combined static PPP position differences between the two models over 1 month. The biggest position difference is less than 1 mm and most points have position differences of less than 0.1 mm. Figure 6 shows the magnitude distribution of these coordinate differences in the North, East, and Up component. The mean biases are −0.6, −2.5 and 0.4 µm and RMS are 1.1, 3.9, and 1.9 µm for each coordinate component. In addition, about 91.5% in the North, 96.9% in the East, and 98.7% in the Up component of all deviations are within twice the standard deviations. These results verify the same position estimates of these two models. 

## 4. Parameter Differences between Combined PPP Models

In the following, we compare the station clocks, ambiguities and pseudo-range residuals between the traditional and simplified GPS/BDS combined PPP models. Data used is the kinematic solution of station JFNG on DOY 028, 2014.

### 4.1. Kinematic Coordinate Difference

**Figure 7:**
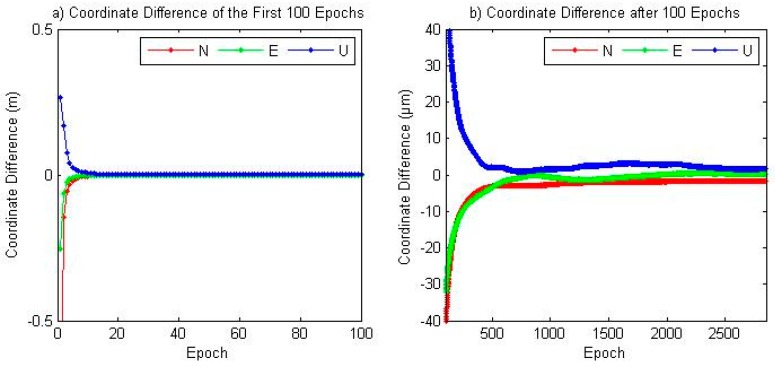
Coordinate Difference between traditional and new model of JFNG at DOY 028, 2014. (**a**) Coordinate difference of the first 100 epochs; (**b**) Coordinate difference after 100 epochs.

As discussed above, in the simplified GPS/BDS PPP model where ISB is not considered, the coordinate solution is indentical with that of traditional model. Here the coordinate difference between traditional and new model of JFNG’s kinematic solution is presented in Figure 7. After 8 epochs the cooridnate differences is less than 1 cm, and after 30 epochs the difference reduces to less than 1 mm. After 500 epochs the difference is less than 5 μm and remains stable until the last epoch.

### 4.2. Station Clock and Ambiguity

**Figure 8:**
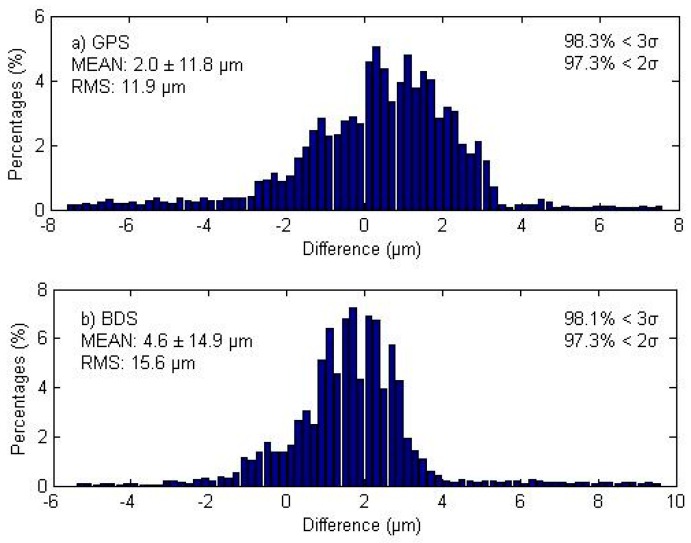
Magnitude distribution of differences between the traditional and simplified models of (**a**) epoch-wise sum of GPS ambiguity and station clock parameters; (**b**) epoch-wise sum of BDS ambiguity, ISB and station clock parameters. Both plots exhibit normal distributions. The top-left corner of each subplot shows the bias and the standard deviation (σ), whereas the top-right corner shows the percentages of deviations that are within 2σ and 3σ.

In the simplified GPS/BDS PPP model, station clock, and ambiguity estimates absorb the ISB parameters together, and the percentage which goes into each parameter depends on the normal equation. In Equation (9), the ISB assimilation shows the opposite sign for station clock and GPS ambiguity parameters, thus the sum quantity of station clock and GPS ambiguity for the traditional and simplified model is theoretically the same at each epoch. The upper subplot of Figure 8 shows the magnitude distribution of this sum quantity differences between the two models using all GPS satellite/station pairs at all epochs. The mean difference is 2.0 µm and all the differences are below 1 mm with around 0.35% bigger than 0.1 mm.

According to Equation (9), the epoch-wise sum of station clock and BDS ambiguity in the simplified model is theoretically the same as the sum quantity of station clock, ISB, and BDS ambiguity in the traditional model. The bottom subplot of Figure 8 shows the magnitude distribution of this sum quantity differences between the two models using all BDS satellite/station pairs at all epochs. The mean difference is 4.6 µm and all the differences are below 1 mm at around 0.72% bigger than 0.1 mm. Both plots show that the differences are so small, which further proves the same coordinate estimates in these two models.

### 4.3. Station Clock and Pseudo-Range Residual

**Figure 9:**
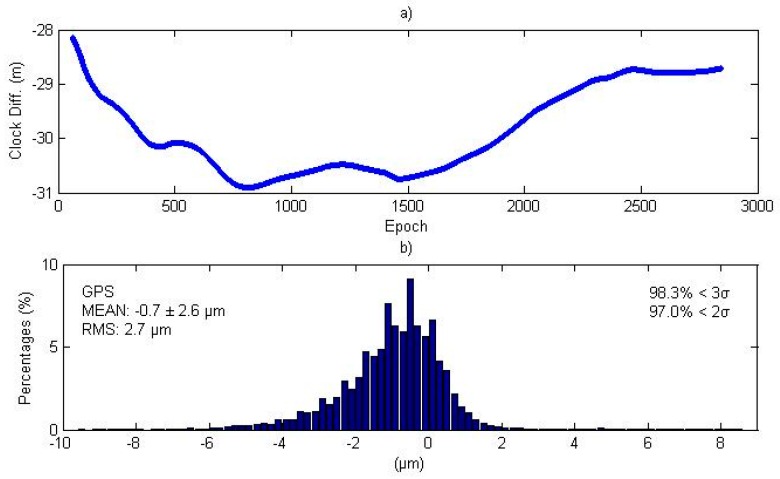
(**a**) Station clock differences between traditional and simplified models; (**b**) Magnitude distribution of differences of the epoch-wise sum of station clock and GPS pseudo-range residuals between the traditional and simplified models. The top-left corner of bottom subplot shows the bias and the standard deviation (σ), whereas the top-right corner shows the percentages of deviations that are within 2σ and 3σ.

In Equation (9), the ISB assimilation shows opposite signals for station clock and GPS pseudo-range observation residuals, thus the epoch-wise sum quantity of station clock and GPS pseudo-range residual is theoretically the same for the traditional and simplified model. The upper subplot of Figure 9 shows the station clock differences between the traditional and simplified PPP models, where the differences are between the range of −31 and −28 m. The bottom subplot of Figure 9 shows the magnitude distribution of the epoch-wise sum quantity differences between the two models for all BDS satellite/station pairs at all epochs. The mean difference is −0.7 µm and all the differences are below 0.1 mm.

### 4.4. Ambiguity and Pseudo-Range Residual

In Equation (9), the ISB assimilation is the same for ambiguity and pseudo-range observation residual in both the GPS and BDS systems. Thus, the ambiguity differences and pseudo-range residual differences between the two models should theoretically be the same at the same epoch for the traditional and simplified model. In the following, we define the epoch-wise “double difference” term by making difference between the ambiguity difference and pseudo-range residual difference between the two models, which by definition should theoretically be zero.

**Figure 10:**
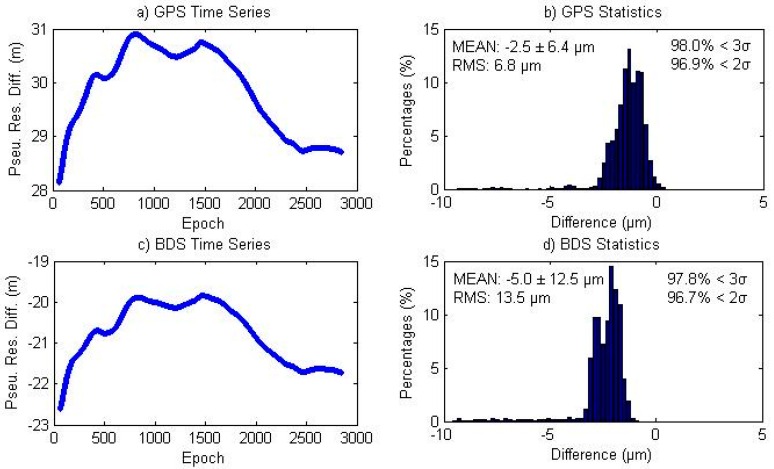
(**a**,**c**) GPS and BDS Pseudo-range residuals between traditional and simplified models; (**b**,**d**) magnitude distribution of the double differences for GPS and BDS observations. The left corner of right subplots (subplot b) and d)) shows the bias and the standard deviation (σ), whereas the right corner of right subplots (subplot b) and d)) shows the percentages of deviations that are within 2σ and 3σ.

The left subplots of Figure 10 show the GPS and BDS pseudo-range residual differences between the two models, where we see that they present a similar shape but with different signs as presented in Equation (9). The right subplot of Figure 10 shows the magnitude distribution of the double difference quantity using all satellite/station pairs at all epochs. The mean double differences are −2.5 and −5.0 µm for GPS and BDS. All the double differences are below 0.1 mm for GPS, while all the BDS double differences are below 1 mm with around 0.5% bigger than 0.1 mm.

## 5. Conclusions and Suggestions

In this study, we discuss the different parameterization of GPS/BDS PPP, where combined PPP between the traditional and a simplified model are compared. GPS/BDS PPP were performed with different models and scenarios using 1 month of GPS/BDS data from 11 IGS MGEX stations. Comparing daily static coordinate estimates of BDS-only, GPS-only and GPS/BDS combined solutions with the IGS daily estimates, the mean 3D RMSes were 3.7, 1.6 and 1.5 cm, respectively. In the kinematic case, the 3D RMSes were 17.0, 8.2, 6.5 cm. Magnitude distribution of the 3D RMS shows higher percentage of more accurate solutions in GPS/BDS combined solutions, which is due to the inclusion of data from different systems.

Comparing GPS/BDS combined PPP coordinate estimates between the traditional and simplified models, mean biases of the differences are −0.6, −2.5, and 0.4 µm and RMSes are 1.1, 3.9, and 1.9 µm in the North, East, and Up components, respectively. The analysis of parameter and pseudo-range residual differences between the two models show the same quantity with deviation at the µm level for: (1) epoch-wise sum of station clock and GPS ambiguity; (2) epoch-wise sum of station clock, ISB, and BDS ambiguity; (3) epoch-wise sum of station clock and GPS pseudo-range residuals; (4) epoch-wise sum of station clock, ISB, and BDS pseudo-range residuals; (5) epoch-wise double difference of ambiguity and pseudo-range residuals. The overall comparisons verify the equivalence of the position estimates derived from the traditional and simplified GPS/BDS combined PPP models.

## Figures and Tables

**Table 1:** Mean RMS of station coordinate differences of different scenarios.

	N (cm)	E (cm)	U (cm)
GPS/BDS	0.34	0.58	1.46
GPS-only	0.36	0.59	1.54
BDS-only	2.09	2.48	2.97
